# Surgical placement of a wireless telemetry device for cardiovascular studies of bovine calves

**DOI:** 10.12688/f1000research.11785.1

**Published:** 2017-07-05

**Authors:** Joseph M. Neary, Vincent Mendenhall, Dixon Santana

**Affiliations:** 1 Department of Animal and Food Sciences, Texas Tech University, Lubbock, TX, 79409-2141, USA; 2 Preclinical Surgery, Clemmons, NC, 27012, USA; 3 Department of Surgery, Texas Tech University Health Science Center, Lubbock, TX, 79430, USA

**Keywords:** biotelemetry, large animal, hemodynamics, cardiopulmonary, waveform

## Abstract

*Background: *Domestic cattle ( *Bos taurus*) are naturally susceptible to hypoxia-induced pulmonary arterial hypertension; consequently, the bovine calf has been used with considerable success as an animal model of the analogous human condition. Studies to date, however, have relied on instantaneous measurements of pressure and cardiac output. Here, we describe the surgical technique for placement of a fully implantable wireless biotelemetry device in a bovine calf for measurement of pulmonary arterial and left ventricular pressures, right ventricular output, and electrocardiogram.

*Methods: *Three, 2-month old bovine calves underwent left-sided thoracotomies. A transit-time flow probe was placed around the pulmonary artery and solid-state pressure catheters inserted into the pulmonary artery and left ventricle. Biopotential leads were secured to the epicardium. The implant body was secured subcutaneously, dorso-caudal to the incision.

*Results: *The implant and sensors were successfully placed in two of the three calves. One calf died from ventricular fibrillation following left ventricular puncture prior to pressure sensor insertion. Anatomical discrepancies meant that either 4 ^th^ or 5 ^th^ rib was removed. The calves recovered quickly with minimal complications that included moderate dyspnea and subcutaneous edema.

*Conclusions: *Left thoracotomy is a viable surgical approach for wireless biotelemetry studies of bovine calf cardiovascular function. The real-time, contemporaneous collection of cardiovascular pressures and output, permits pathophysiological studies in a naturally susceptible, large animal model of pulmonary arterial hypertension.

## Introduction

The bovine calf ( *Bos taurus*) has provided invaluable insight into the structural, mechanical, and hemodynamic changes that occur during the onset and progression of pulmonary arterial hypertension (PAH) ^1– 4^. Hemodynamic studies of calves conducted to date, however, have typically required the calf to be manually restrained while laterally recumbent ^2, 4^.

Fully implantable biotelemetry devices with transit-time volumetric flow measurement capability are available for use in animals. This technology permits real-time, contemporaneous collection of hemodynamic variables without the risks, or potentially confounding effects, associated with the catheterization of calves with compromised cardiac function. Animal welfare is also improved for two major reasons: first, cardiac function and vascular pressures can be closely monitored so that experimental and humane endpoints can be established using cardiac output and pressure variables, and second, invasive procedures are limited to the initial surgery performed prior to the onset of pulmonary hypertension. In this report, we describe a surgical technique for the placement of biotelemetry equipment in a calf model of PAH.

## Methods

All procedures described in this study were pre-approved by the Texas Tech University Animal Care and Use Committee (Protocol 16024-03). All efforts were made to ameliorate any suffering experienced by the animals in the study by monitoring the effectiveness of the analgesics used throughout the study and combining analgesics that had complementary mechanisms of action.

### Preoperative care

Three male, castrated Jersey calves, weighing between 47 and 54 kg, were obtained from a dairy in West Texas. Calves were collected at 2-months of age. At this age, calves are less susceptible to gastrointestinal pathogens than neonatal calves and less susceptible to respiratory pathogens than older calves with waning colostrum-derived maternal antibodies ^5, 6^.

Calves were fed 4 to 5-liters of colostrum within 24-hours of birth. They were subsequently fed 12–14% of their body weight per day with calf milk replacer reconstituted in warm water (0.15 kg at ≥ 22% crude protein on a dry matter basis per 1 L of water). Calves were fed milk replacer twice per day until 6-weeks of age when they were fed once per day. From 2-weeks of age they were provided with *ad libitum* access to a pelleted complete ration calf starter (≥ 20% crude protein, dry matter basis). Calves were individually housed on a raised slatted floor to reduce pathogen transmission and soiling of the calves’ coats. Halter training was performed over several days commencing one day after arrival from the dairy farm. In brief, training involved accustoming calves to human touch, the feel of an adjustable nylon-rope head halter, being led, and standing still.

Calves were fasted for 12-hours prior to surgery. One-hour prior to surgery, a 16-gauge, 2” (5 cm) catheter was placed in jugular vein. Intradermal lidocaine (2%) provided local analgesia. Pre-operative medications were then given. These included the broad-spectrum antibiotic ceftiofur sodium (2 mg/kg iv; Naxcel, Zoetis, Parsippany, NJ, USA) and the non-steroidal anti-inflammatory drug meloxicam (0.1 mg/kg iv; Metacam, BoehringerIngelheim, Vetmedica, Inc., Duluth, GA, USA). The area between the pre-scapular region to the 10 ^th^ rib, and from the dorsum to the sternum was clipped on both sides of the chest.

### Sizing of the transit-time Doppler flow probe

Perivascular cuffs with 3 cm internal diameters were used for flow measurements. Correct sizing was determined prior to starting this study from echocardiographic measurements of mainstem pulmonary arterial diameters of 2-month old Jersey calves (n = 5) (Vivid i and 3S-RS 2.0 to 3.6 MHz phased array transducer probe; General Electric, Fairfield, CT, USA).

### Anesthesia and intubation

Calves were induced using a combination of diazepam (0.25 mg/kg iv), ketamine (5 mg/kg iv), and buprenorphine (0.005 mg/kg iv). Following induction, calves were intubated with a cuffed silicone Murphy eye endotracheal tube (10mm ID) and maintained on isoflurane for the duration of surgery. The isoflurane vapor was set at 5% at induction with oxygen flow at 20 mL/kg/min. This was subsequently reduced to between 0.5–3% with oxygen flow at 10 mL/kg/min. Flow rates and vapor settings were adjusted according to the plane of anesthesia. Calves were ventilated to effect using positive-pressure ventilation (approximately 400–600 mL tidal volume at 8–12 breaths/min, 18–20 cm H _2_O; SAV 2500, SurgiVet, Smiths Medical, Dublin, OH, USA). Lactated ringers containing 5% dextrose was provided at 300 to 400 mL/h (9 mL/kg/h iv).

Physiologic measurements collected every 5-minutes included body temperature (rectal thermometer), end-tidal CO _2_ and breathing rate, heart rate, oxyhemoglobin saturation (pulse oximeter on tongue), capillary refill time, mucous membrane color, and non-invasive arterial blood pressures (antebrachial cuff). Arterial blood was collected from a 20-gauge 1” (2.5 cm) catheter in either the auricular or brachial artery every 60-minutes and analyzed on portable blood-gas analyzer (VetScan i-STAT 1, Abaxis, Union City, CA, USA).The paralytic agent atracurium (0.4 mg/kg iv) was administered once the calf was connected to the positive-pressure ventilator and prior to the initiation of surgery. A reversal agent, neostigmine, was available, but not used.

### Surgical procedure

Prior to the initiation of surgery, intercostal nerve blocks were performed by injecting 3 mL of lidocaine (2%) in the dorsal aspect of the third, fourth, and fifth intercostal spaces. Lidocaine was injected subcutaneously along the fourth intercostal space. The skin was incised along the fourth intercostal space (#15 blade). A left anterolateral thoracotomy through the fourth intercostal space was performed using electrosurgery (Bovie 2350-V, Bovie Medical Corporation, Purchase, NY, USA). Power settings of 30 W for both cut and coagulation modes were used. With a periosteal elevator, the entire length of the rib periosteum was elevated and transected anteriorly and posteriorly with rib cutters. The 5 ^th^ rib was removed from the first two calves and the 4 ^th^ rib was removed from the third calf. Finochetto retractors (33 cm spread, blades: 19 cm long and 5 cm deep) were placed to improve cardiac access. Next, the pericardium was opened and stay sutures placed to retract it away from the surgical field. Care was taken to retract the phrenic and vagal nerves with stay sutures. These nerves cross the left lateral aspect of the mainstem pulmonary artery.

**Figure 1.**
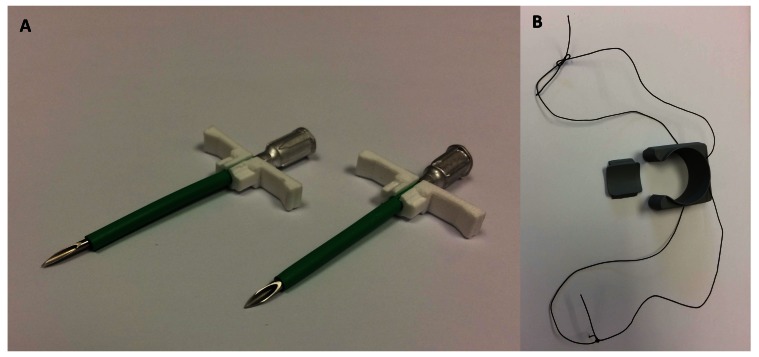
Equipment used for biotelemetry device placement. ( **A**) In the first two surgeries, a 16-gauge, 5 cm needle served as a stylet inside a splittable introducer (left). A 14-gauge 5 cm needle was used as a stylet inside a beveled introducer in the third surgery (right). ( **B**) 3.5 Metric braided silk suture was inserted through two eyelets located on the lateral aspects of the flow probe perivascular cuff creating two loops of suture.

First, a solid-state pressure sensor was placed in the apex of the left ventricle. To do this, the apex of the heart was manually elevated and a purse-string suture placed using 1.5 or 2.0 Metric polypropylene suture on a double-armed, taper point needle. The cardiac apex was manually elevated according to hemodynamic tolerance. The heart was intermittently lowered back into the chest to avoid severe hypotension and bradycardia. In the first two surgeries, a 16-gauge, 5 cm needle served as a stylet inside a splittable introducer (EG-ACC-PID7F, Transonic EndoGear Inc, Ithaca, NY, USA). A 14-gauge 5 cm needle was used as a stylet in the third surgery ( Figure 1A). After placement of the introducer in the apical wall, the stylet was removed and the pressure sensor inserted. The introducer was then peeled apart to leave the pressure sensor embedded within the myocardium. The purse-string was tightened and the sensor secured using a Chinese finger-trap suture pattern (1.5 or 2.0 Metric polypropylene suture). A 2% lidocaine infusion was given (1mg/kg slow iv) during the third surgery prior to manipulation of the heart or pulmonary artery to reduce the risk of dysrhythmias.

Next, one of the two biopotential leads was placed adjacent to the left ventricular pressure sensor. A 20-gauge, 2.5 cm needle served as a channel in the myocardium, through which 2 to 3 cm of the lead was fed. The needle was removed leaving approximately 1 cm of the biopotential lead embedded in the myocardial wall. The free end of the lead was secured in 2 to 3 places using 1.5 or 2.0 Metric polypropylene suture in a simple interrupted pattern. Arterial blood pressure was closely monitored while the cardiac apex was manually elevated.

**Figure 2.**
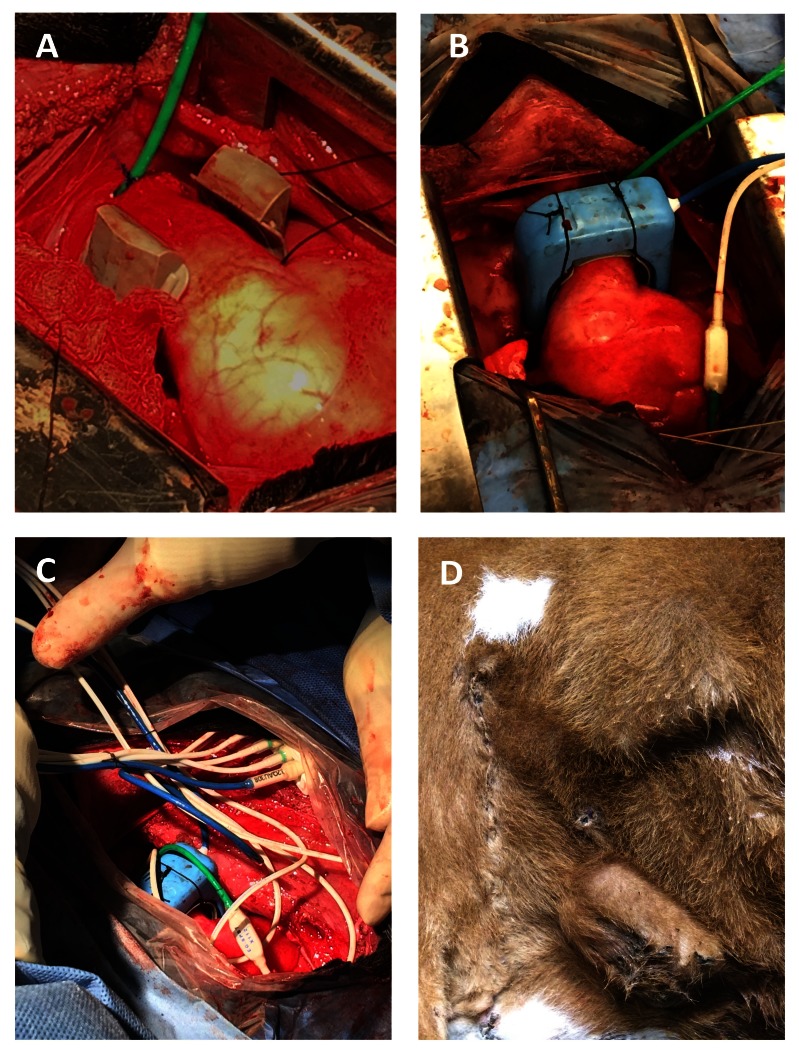
Biotelemetry implantation via a left thoracotomy. ( **A**) Loops of silk suture, originating from the lateral aspect of the perivascular cuff, pass on each side of the mainstem pulmonary artery. A pressure sensor (green) has been inserted into the pulmonary artery distal to the perivascular flow probe cuff. ( **B**) The flow probe was then secured to the perivascular cuff using loops of suture previously placed through cuff eyelets. ( **C**) Telemetry leads were bundled together and secured with 3.5 Metric braided silk suture. ( **D**) The implant body and battery were placed in subcutaneous pockets dorso-caudal and ventro-caudal to the thoracotomy incision, respectively.

The mainstem pulmonary artery was freed from connective tissue using a combination of sharp dissection and electrosurgery. Prior to placement of the perivascular flow probe cuff, threads of 3.5 Metric braided silk suture were placed through two eyelets located on the lateral aspects of the cuff base (as it appears in Figure 1B). This created two separate loops of suture so that following placement of the cuff around the base of the mainstem pulmonary artery, one loop passed on the cranial side of the pulmonary artery and the other passed on the caudal side ( Figure 2A). Next, a purse-string suture (2.0 Metric polypropylene) was placed in the pulmonary artery wall distal to the flow probe and 2 to 3 cm proximal to the main trunk arterial bifurcation. The arterial wall in the center of the purse string was punctured with a scalpel blade (#11) and a second solid-state pressure sensor inserted. The sensor was secured using a Chinese finger trap pattern, as previously described ( Figure 2A). The flowprobe (EG-32QAU-X Transonic EndoGear Inc) was then secured to the perivascular cuff using the loops of suture previously placed through eyelets in the cuff ( Figure 2B). The second biopotential lead was secured to the right auricle in the first calf. To avoid the minor bleeding experienced in the first calf, the lead was embedded within the atrioventricular myocardium of the third calf.

To facilitate implant removal, telemetry leads were bundled together and secured with 3.5 Metric braided silk suture ( Figure 2C). A subcutaneous pocket was created dorso-caudal to the thoracotomy incision where the telemetry implant (EG2-Q1S3tM25, Transonic EndoGear Inc), enclosed in a polypropylene mesh, was placed so that the antenna was dorsally located. The battery was placed in a subcutaneous pocket, ventro-caudal to the thoracotomy incision ( Figure 2D).

Two to four cable ties were used to approximate the thoracotomy incision and suture the muscles in an anatomical fashion. Hemostats were used to pull cable ties through the intercostal muscles. Once all cable ties were placed, towel clamps were used to grasp the ribs and minimize the thoracotomy incision, while the second surgeon tightened each cable in an alternating pattern until adequate closure was obtained.

The chest wall was closed in two layers using 3.5 Metric polydioxanone suture in a simple continuous pattern. The skin was closed with a running subcuticular pattern (3.5 Metric polydioxanone suture). Time from first incision to skin closure was 3.5 hours. Anesthesia recovery was uneventful. The calves walked back to the pen 30-minutes after turning the isoflurane vapor to 0%.

A 42 cm long 30 Fr chest tube (7 mm ID, 10 mm OD) with side holes was used to remove air from the chest cavity following skin closure. The tube was placed within the chest cavity prior to thoracotomy incision reduction with cable ties. A Valsalva maneuver was performed to decompress the free intrathoracic air, expand the lung, and avoid a tension pneumothorax. The external end of the tube was submerged in saline throughout this procedure.

### Postoperative recovery

Meloxicam (0.1mg/kg sid iv) and buprenorphine (0.005 mg/kg q 8h iv) were provided for post-operative pain relief. Ceftiofur sodium (1mg/kg sid iv) was continued for 2-days post-surgery.

## Results

Surgeries were performed on August 23 ^rd^ and 24 ^th^ (JMN and VM) and December 5 ^th^, 2016 (JMN and DS). The first and third surgeries were performed with minimal complications. The second calf, however, died from ventricular fibrillation following puncture of the left ventricular apex prior to pressure sensor placement. The splittable introducer sheathing the 16-gauge needle appeared to snag on the myocardium as it was inserted through the ventricular wall. This may have been attributable to the loose fit ( Figure 1A). A 14-gauge was subsequently found to be a more appropriate fit for the introducer and was used as a stylet in the third surgery. The introducer used in the third surgery was also beveled at the tip to ease tissue passage ( Figure 1A). A cardiac defibrillator was not available; consequently, intracardiac and intravenous epinephrine were administered to correct the dysrhythmia, but to no effect. Lidocaine (2%) was intravenously infused prior to cardiac manipulation in the third calf to reduce the risk of dysrhythmias.

Calves were stable throughout the surgeries. The greatest variations in heart rate (70 to 250 beats per minute) and arterial blood pressure (100/77 to 154/125, systolic/diastolic) were observed during the first surgery. Arterial oxyhemoglobin saturations were ≥ 88% and typically > 95%. Body temperatures decreased by 2°C by the end of the surgeries.

### Surgical complications

The 5 ^th^ rib was the most appropriate rib to remove during the first surgery. This optimized access to the pulmonary artery and cardiac apex. The 5 ^th^ rib was also removed during the second surgery, but, in hindsight, removal of the 4 ^th^ rib would have been preferable. Because of these anatomic discrepancies, access to the left ventricle and pulmonary artery was carefully appraised during the third surgery so that the most appropriate rib – in this case, the 4 ^th^ rib – was removed.

### Postoperative recovery

The first calf presented with mildly labored breathing and diminished lung sounds over the left chest the morning after surgery. Normal lung sounds and breathing were restored following 2-days of furosemide (4mg/kg bid iv). The third calf developed moderate subcutaneous edema around the implant body and battery 3-days after surgery, which resolved 2-days later without treatment. Calves resumed a normal appetite within 3-days of surgery. Calves remained healthy until the end of the study 18-days (Calf 1) and 17-days (Calf 3) post-surgery.

### Data acquisition

**Figure 3.**
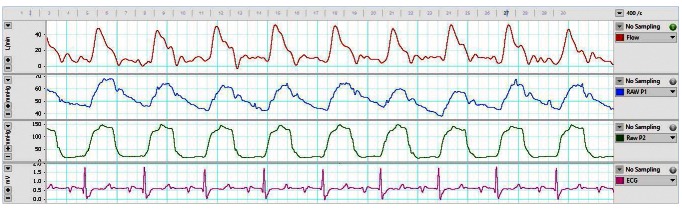
Example telemetry data from calf 3 approximately 3-hours post-surgery. Pulmonary arterial flow (L/min, top channel), pulmonary arterial pressure (mmHg, second channel), left ventricular pressure (mmHg, third channel), and electrocardiogram (ECG)(mV, bottom channel) over a 5-second period from a 2-month old Jersey calf one-day post-surgery.

Although the perivascular cuff appeared loose during surgery, transit-time volume flow measurement stabilized approximately 6-hours (calf 1; see data file: Calf 1 – Day of surgery ^7^) and 3-hours (calf 3; see data file: Calf 3 – Day of surgery ^7^) following the completion of surgery ( Figure 3). However, it was not until approximately 4 days post-surgery that flow measurements became consistent between readings (Data files: Calf 1 – 4-days post-surgery, and Calf 3 – 4-days post-surgery ^7^). Electrocardiogram recordings became sporadic on the 4 ^th^-day (Calf 1) and 10 ^th^-day (Calf 3) post-surgery and ceased altogether the following day in both calves.

## Discussion

The results of this study indicate that a left thoracotomy is a viable surgical approach for wireless biotelemetry studies of bovine calf cardiovascular function. The contemporaneous, real-time collection of cardiovascular pressures and transit-time volume flow in a bovine model of pulmonary arterial hypertension permits detailed pathophysiological studies of hemodynamically complex disease processes such as pulmonary arterial hypertension.

An important consideration prior to the initiation of a biotelemetry study involving transit-time volume flow measurement is selection of an appropriately sized perivascular cuff. In our study, the mainstem pulmonary artery of 2-month old calves was measured by echocardiography. Vessel diameter measurement from cadavers underestimates the true *in vivo* vessel size as the effects of intravascular and intrathoracic pressures on vessel distension are ignored.

The calves in our study quickly recovered from surgery and showed minimal evidence of post-operative pain; consequently, only a short recovery period of ≤ 5 days, was necessary prior to the initiation of the subsequent study. The calves were also halter-trained prior to surgery to minimize the stress associated with animal handling. Stress is deleterious to wound healing and surgical recovery ^8^; consequently, the recovery period may have been longer had the calves not been halter-trained. Another benefit of halter training is that procedures, such as echocardiography, can be performed on a relaxed, standing animal. This minimizes any stress-induced perturbations of the acquired data.

This study was limited by the small number of animals studied. We have, however, successfully described a surgical technique for the placement of a fully implantable wireless biotelemetry device in a bovine calf for measurement of pulmonary arterial and left ventricular pressures, right ventricular output, and electrocardiogram. The techniques described in this study will likely be refined in future studies.

In conclusion, left thoracotomy is a viable surgical approach for wireless biotelemetry studies of bovine calf cardiovascular function. Successful surgical implantation and application of this technology has considerable potential to advance our understanding of the hemodynamic changes that occur during the onset and progression of pulmonary arterial hypertension in a naturally susceptible bovine animal model.

## Data availability

Telemetry data files for calves 1 and 3 are available at http://dx.doi.org/10.7910/DVN/N2US1Y ^7^.
