# Supplementary material for: Surgical placement of a wireless telemetry device for cardiovascular studies of bovine calves
Source: F1000Res. 2017 Jul 5;6:1061. [Version 1] doi: 10.12688/f1000research.11785.1 (PMC5790998; doi:10.12688/f1000research.11785.1)
Supplement: Peer review history

# Peer review: Referee response for version 1

**DOI:** 10.5256/f1000research.12731.r28615

**Published:** 2018-01-29

**Authors:** Susana Astiz

**Affiliations:** 1 National Institute for Agricultural and Food Research and Technology (INIA), Madrid, Spain

It is an innovating work, well written and accurately described that describes a (short-term) successful surgical technique to implant a wireless telemetry device in calves.

 I just miss a bit more “general” information about arterial hypertension studies to have an idea on how long should such studies be, and to justify the election of large animal models. On the other side, this study has to be understood as preliminary work and results, with several issues susceptible to be improved. Specific suggestions would be, the reduction of the size of the devices to be implanted, in order to make possible the use of minimally invasive surgery, and in order to avoid, mainly, any rib resection.

 Other more specific comments are the following:

 Preorperative care, first paragraph; the age of the calves 82 m old) are justify with a reference (the 6 ^th^). I suggest to take the following reference instead of or additive to the 6 ^th^: The median age stated in this very recent study for respiratory problems in dairy calves is 29-60 days of age. ^1^

 Regarding the following paragraph of this section, a very detailed description is written about how the calves were managed during the time before arriving at the hospital. However, it would be more useful to know how long the calves were at the hospital before the surgeries were performed. Days? weeks? And information about how they were managed at the hospital the days before and after the surgery (feeding, m2/animal, individual housing or not…). How long did the halter training take?

 Paragraph “Postoperative recovery”; It is stated that one administration of meloxicam was given. I would rather advise to repeat the meloxicam treatment once more (every three or two days, at least twice), such that the analgesia is assured for longer time, specifically when a rib had to be resected.

 Results section; In this section, under the Postoperative recovery subsection, it is stated that “Calves remained healthy until the end of the study 18-days (Calf 1) and 17-days (Calf 3) post-surgery.” Did the calves die after those time periods? Or were they still alive? Could we have this information? I do ignore how long a cardiovascular study hypoxia-induced pulmonary arterial hypertension should ideally last, but to know how long those calves live after the surgery, would be a relevant information to consider performing this surgery by other research groups.

 Finally, a more detailed description on how the calves recovered (not only intake, but feeding and social behavior, vocalizations, pain score, etc.)  is desireble.

I have read this submission. I believe that I have an appropriate level of expertise to confirm that it is of an acceptable scientific standard.

## References

[1] Medrano-GalarzaCLeBlancSJJones-BittonADeVriesTJRushenJMarie de PassilléAEndresMIHaleyDB: Associations between management practices and within-pen prevalence of calf diarrhea and respiratory disease on dairy farms using automated milk feeders. *J Dairy Sci*.2017; 10.3168/jds.2017-13733 10.3168/jds.2017-13733 29290433

# Peer review: Referee response for version 1

**DOI:** 10.5256/f1000research.12731.r24047

**Published:** 2017-07-12

**Authors:** Guillaume Chanoit

**Affiliations:** 1 School of Veterinary Sciences, University of Bristol, Bristol, UK

This article is nicely written and the methodology is clearly explained. There is a clear rationale to develop the technique that the authors are presenting. The telemetry techniques used in cardiovascular research on animals are contributing to animal welfare (reduction and refinement alternatives), and are reducing overall animal research costs. Some articles describe the technique in small animal models but very few examples of its use in large animals are available in the literature.

 As the paper only includes 3 cases, one of which died before data acquisition, this report should be considered as the very early learning curve of the technique presented.

 The surgical approach described included a rib resection to allow sufficient exposure to the structures of interest. I wonder if performing two thoracotomies (in the 3 ^rd^ and 4 ^th^ or 4 ^th^ and 5 ^th^ intercostal spaces) would  maybe alleviate the need for a rib resection, which is known to be a potentially painful procedure. It may also be possible to consider an hybrid approach that would allow a device to be implanted in the pulmonary artery (PA) using right heart catheterization for PA pressure measurement combined with minimally invasive surgical placement of the bipotential leads and pressure sensor.

 The animals were given buprenorphine postoperatively. Have they been pain scored to determine that buprenorphine was sufficient and that stronger opioids were not needed? Calves are considered pray animals and are therefore expressing pain a more concealed manner which may give an initial false impression that their pain level is low.

 It would be interesting to know how long the authors think the devices surgically placed will last in a growing calf. Working with other animal models (such as pig) the growth of the animal has to be taken in consideration for any chronic study- I presume it would be the same for a study in calves?

I have read this submission. I believe that I have an appropriate level of expertise to confirm that it is of an acceptable scientific standard.
